# Medical Clinic

**Published:** 1883-04

**Authors:** Alfred L. Loomis

**Affiliations:** Professor of Pathology and the Practice of Medicine in the Medical Department of the University of the City of New York, held in Bellevue Hospital


For Contents See Page 248.

THE

### Independent Practitioner.

Vol. IV. April, 1883. No. 4.

### Original Communicatons.

We are not responsible for the opinions expressed by contributors.

Article I.

### MEDICAL CLINIC.

[Concluded from March Number.]

BY ALFRED L. LOOMIS, A.M., M.D.,
Professor of Pathology and the Practice of Medicine in the Medical Department of the University of the City of New York, held in Bellevue Hospital.

CASE II.

### Pleurisy, Acute Bronchitis and Bronchial Hemorrhage.—A Question of Phthisical Developments Subsequent.

*Gentlemen* :—This young woman, æt 18 years, says that she was perfectly well up to two years ago, when she was taken with a severe pain in her left side. The pain was so severe that it prevented a full inspiration. Her sickness kept her in bed for about one week, but she remained ill for six months after.

Her physician told her that she had pleurisy. She does not remember whether or not she had a chill at the commencement of the disease. Her chief symptoms were *shortness of breath* and *pain in the side.* From that sickness she says she entirely recovered, so that she felt perfectly well, having neither cough nor pain, and being able to lie upon one side as well as the other.

She remained well until about four weeks ago when, as she says, she " began to feel all sore on the inside on both sides of the chest, but more on the left side than on the right side."

She called a physician, who applied a blister to the left side, and she was so far relieved that she was able to be up again, but not able to attend to her occupation, which is that of a silk weaver.

While she had the "soreness in the chest," she had some cough, which has continued, and is attended by the expectoration of a considerable quantity of muco-purulent material.

About three weeks ago she began to "raise blood," at first five or six mouthfuls of thick, but not very dark blood, and, she says, it looked very much "like a clot."

On the following day she again expectorated about the same quantity of blood. The day following, she says she vomited about two quarts of blood, the vomiting being preceded by a paroxysm of coughing. Hæmatemesis continued only for about one week, and since that time she has continued to cough, and to expectorate quite freely "yellowish phlegm." During the past four weeks she has lost flesh. At the present time she has a chilly sensation in the morning, some fever towards the close of the day, and occasional sweating in the night. Her pulse is 76; temperature is 99°. There is no history of phthisis in her family.

The question arises in these cases: is there any connection between the pleurisy which she suffered two years ago and her present condition? Is a subacute pleurisy ever the remote starting point of a phthisical development? I am more careful with respect to the after treatment of one who has had an attack of subacute pleurisy than I am with reference to the after treatment of a case of acute pneumonia. If a man recovers from pneumonia, his recovery is complete, and thereafter he is as well as ever he was, unless, at the same time, he had an extensive pleurisy.

One who has had subacute pleurisy may get entirely well, but if he has any phthisical tendency, thickening of the pleura is quite sure to be followed sooner or later by pulmonary disease.

Now, making a physical examination, we notice on inspection, that there is a slight depression under the clavicle on both sides of the chest. *On percussion* we find normal resonance upon the right side under the clavicle, but upon the left side the pitch is higher. This difference in the percussion note is shown better by light than by heavy percussion, and this is very good evidence that whatever interferes with normal resonance is quite superficial. Strong percussion brings out the deep-seated resonance. There is then slight dulness under the left clavicle recognized by light percussion. On percussion the chest *posteriorly* we find dulness upon the left sides, as compared with the right, and in the scapular region there is almost complete flatness.

Expansion of the chest is very good upon both sides.

Vocal fremitus does not tell us anything in this case, since the patient's voice is normally high-pitched, and there exists some laryngeal trouble which causes almost complete aphonia.

The result of the physical examination thus far is, general dulness over the entire left side of the chest, most marked beneath the scapulæ, but also well marked below the clavicle. The dulness is more marked at the upper than at the lower portion of the left side of the chest.

*On auscultation*, exaggerated respiration is heard all over the right side of the chest, and its character indicates that it is *not* of recent origin. In other words, I am led to believe from the character of the respiration that the right lung has for a long time being performing extra work. The vesicular murmur is feeble over the entire left side of the chest.

Pleuritic creakings and crepitations are heard in front and behind, but are most marked just under the scapula. The respiratory murmur can be heard at the bottom of the chest, although it is feeble there.

Inspiration is interrupted and " wavy " in character.

Mucous râles of small size are heard in abundance in the supra-scapular region, and a few bronchial râles can be heard in front.

The only place at which the respiration has anything like a bronchial character, is just at the border of the supra-scapular fossa, and underneath the scapula. There is, then, only slight evidence of lung consolidation from these physical signs. We reach the diagnosis that there are thickenings of the pleura and adhesions over the entire left side of the chest. The physical signs which indicate this are most marked in the lower portion of the chest cavity.

In addition to this, we hear rude respiration over the entire left side of the chest, but although rude, it is diminished in intensity.

Underneath the scapula there is some bronchial *expiration*, but *not* bronchial breathing.

Mucous râles, almost subcrepitant, are heard in the supra-scapular fossa in abundance, and less abundant under the clavicle upon the left side.

There has either been a thickening of the mucous membrane of the bronchial tubes, or they contain a tenacious secretion which gives rise to imperfect sonorous râles. It may be that the change which gives rise to this sound is in the *larynx*, for when laryngeal changes have gone to the extent of producing nearly complete aphonia, it is sometimes very difficult to determine the presence of bronchial respiration. In other words, you will think that you hear bronchial respiration in lungs wherein there is no consolidation. I have, in a number of instances, noticed that it was difficult to determine where bronchial and amphoric respiration was present when changes in the larynx co-existed. In cases of advanced phthisis, examination of the chest is rendered more or less unsatisfactory by the existence of these laryngeal changes.

I believe that this woman had an acute pleurisy, which was followed by a sub-acute pleurisy, with a large amount of serous effusion, containing considerable fibrinous material, and that it was six months before she recovered sufficiently to be able to go about her work ; that during that time firm adhesions formed between the two surfaces of the pleura upon the left side. Because of the pleuritic adhesions, nothing would more disturb the circulation within the chest than an acute attack of bronchitis. As a result of such disturbance hyperæmia occurred, and a more or less profuse bronchial hemorrhage followed.

Ordinarily such an attack of bronchitis would be attended simply by a muco-purulent expectoration ; but, with pleuritic adhesions, what would otherwise have been a simple bronchitis is changed into a bronchitis attended by hemorrhage.

The fact that there was a bronchial hemorrhage, is evidence that there was a disturbance in the pulmonary circulation by the adhesions from the pleurisy.

As a result of the acute attack of bronchitis and derangement of pulmonary circulation, she has reached a condition in which there is evidence of *slight* consolidation of the lung beneath the scapul. Whether this slight consolidation developed in connection with the old pleurisy, or whether it is of recent origin, cannot be decided. In order to settle that question it will be necessary to watch the case for some time. If it is found that the evidence of pulmonary consolidation disappears, it must be regarded as of *recent* origin. I am inclined to believe, however, that it is an old condition, and that she has

*no* real catarrhal pneumonia, but that there are fibrous changes in the lung, the result of her attack of pleurisy. In other words, although she is not yet in the condition in which one can say she has fibrous phthisis (for fibrous phthisis cannot be positively diagnosticated until there is evidence of contraction of the chest walls together with evidence of pulmonary consolidation), yet she is in a condition in which you are quite certain to see fibrous or catarrhal phthisis sooner or later develop.

If she becomes debilitated by bronchial hemorrhages, the bronchitis, which appeared as an acute disease, will almost certainly lead to broncho-pneumonia and catarrhal phthisis. If, however, she recovers from the bronchitis and does not have a renewal of the pleurisy (and this is quite possible), she will nevertheless be in as great danger as she has been for two years past. For, whenever she has an acute pulmonary affection—no matter what it may be—she will be very liable to develop phthisis, and most probably the catarrhal variety.

If instead of being free from hereditary taint, she possesses an inherited tendency to phthisis, these pleuritic changes which were fibrous in their nature and non-tubercular at the commencement, would be followed in all probability by a phthisis that would be both tubercular and fibroid. And, in saying this, I mean that if tubercles are developed, it would be from an inherited tendency, a "vice of blood" or diathesis. The treatment is to be stimulating and nourishing. A change of climate would be of very great benefit.

---

According to Les Mondes (Med. and Surg. Rep.), it appears that in Russia, France and England, consumption of tobacco amounts to one pound per inhabitant. In Italy it is higher, one and a half pounds. Austria comes next with two and two-fifths pounds. In the United States and Germany the consumption amounts to three pounds, in Belgium four and four-fifths pounds, while Holland has the privilege of heading the list with a consumption per inhabitant of more than five and a half pounds.—*Exchange.*

